# Social Presence and Use of Internet-Delivered Interventions: A Multi-Method Approach

**DOI:** 10.1371/journal.pone.0057067

**Published:** 2013-02-20

**Authors:** Rik Crutzen, Dianne Cyr, Hector Larios, Robert A. C. Ruiter, Nanne K. de Vries

**Affiliations:** 1 Department of Health Promotion, Maastricht University, Maastricht, The Netherlands; 2 Beedie School of Business, Simon Fraser University, Vancouver, BC, Canada; 3 School of Interactive Arts and Technology, Simon Fraser University, Vancouver, BC, Canada; 4 Department of Work and Social Psychology, Maastricht University, Maastricht, The Netherlands; CSIC-Univ Miguel Hernandez, Spain

## Abstract

**Objective:**

Internet-delivered interventions can effectively change health risk behaviors and their determinants, but adherence to intervention websites once they are accessed is very low. This study tests whether and how social presence elements can increase website use.

**Methods:**

A website about Hepatitis A, B, and C virus infections was used in a preparatory lab-based eye-tracking study assessing whether social presence elements attract participants' attention, because this is a prerequisite for affecting website use. In the following field study, 482 participants representative of the Dutch population were randomized to either a website with or a website without social presence elements. Participants completed a questionnaire of validated measures regarding user perceptions immediately after exposure to the website. Server registrations were used to assess website use.

**Results:**

Participants in the experimental condition focused on the social presence elements, both in terms of frequency (*F*(1, 98) = 40.34, *p*<.001) and duration (*F*(1, 88) = 39.99, *p*<.001), but did not differ in website use in comparison with the control condition; neither in terms of the number of pages visited (*t*(456) = 1.44, *p* = .15), nor in terms of time on the website (*t*(456) = 0.01, *p* = .99).

**Conclusions:**

Adding social presence elements did not affect actual use of an intervention website within a public health context. Possible reasons are limited attention for these elements in comparison with the main text and the utilitarian value of intervention websites.

## Introduction

Internet-delivered interventions can effectively change health risk behaviors and their determinants [1], but the actual use of these interventions by the target group once they access the website is very low [2]. Eysenbach [3] even defined the “law of attrition”, which considers high attrition rates as a natural and typical feature of Internet-delivered interventions. For example, server statistics of an intervention promoting heart-healthy behaviors showed that 285,146 visitors from unique IP addresses accessed the home page in a 36-month period, but 56.3% of them left the intervention website within 30 seconds [4]. This finding touches upon a critical issue in Internet-delivered interventions: how can they ever have a public health impact if people only briefly use the actual intervention? It is therefore relevant and necessary to focus on factors related to the use of an Internet-delivered intervention once people arrive at the intervention website (i.e., website use) [5]. These factors relate to the *visitor* (e.g., people's motivation to be healthy [6], [7]) as well as the *intervention* website (e.g., offering tailored information [8], [9]). Not only is the content of the website important [10], but also the specific characteristics of the website itself. The current study aims to systematically manipulate a website characteristic – i.e., social presence – and link this to (1) website use and (2) user perceptions as the working mechanisms behind this possible effect of social presence. The rationale behind this aim is explained in the follow paragraphs, resulting in hypotheses to be tested in the current study.

The focus of the current study is on social presence, which can be seen as the extent to which a medium is perceived to convey a feeling of human contact, sociability, and sensitivity [11]. This is realized by adding social presence elements: presenting human images [12] and testimonials [13] in which people share their experiences and ideas regarding the information presented on the website with others. Previous studies revealed that adding these social presence elements increases perceived social presence and positively affects attitude towards shopping websites [14] as well as intention to use them [13]. Two issues remain unclear, however. First, whether social presence also has added value in a public health context. Second, whether the positive effect regarding attitude and intention to use also hold when looking at the actual behavior (i.e., website use) instead of its precursors. Given that intention is the most important predictor of actual behavior [15], this is hypothesized to be the case. Thus, social presence is expected to increase website use in a public health context (Hypothesis 1).

What are the working mechanisms behind this possible effect of social presence on website use? The main idea is that social presence results in a positive user experience, which increases website use. User experience refers to what a person thinks and feels during and after being exposed to a website [16] and is deemed even more important than usability, which only concerns ease of use [17]. User experience consists of both cognitive perceptions (e.g., efficiency, effectiveness) and affective perceptions (e.g., enjoyment) [18]. *Efficiency* refers to easy search and access of information provided and *effectiveness* refers to the quality of that information (e.g., in terms of relevance) [19]. These cognitive perceptions have parallels with perceived ease of use and perceived usefulness in the technology acceptance model, but are applicable in a broader context [20]. The affective perceptions of user experience are often referred to as *enjoyment* [21], [22]. *Active trust* refers to the confidence in acting on the provided information on a website, which can result in increased website use [23]. Efficiency, effectiveness and enjoyment can be expected to be positively associated with website use and these associations are hypothesized to be partly mediated by active trust, which is in line with the limited evidence that is currently available [24], [25] (Hypothesis 2).

Previous studies investigating social presence and user perceptions consistently demonstrated that social presence positively affects perceptions of both effectiveness and enjoyment regarding shopping websites [13], [14], [26]. The current study extends previous studies by including a measure of *interest* besides enjoyment, because this is a motivational factor that “guarantees the person's engagement” [27]. Although interest is sometimes defined as feelings of enjoyment [28], interest and enjoyment differ in critical ways [29]. Experiments using several kinds of stimuli (e.g., random polygons, poetry, and experimental paintings) indicate that enjoyment is not a necessary condition for interest [30]. The two antecedents of interest are, according to Silvia [29], appraisal of coping potential (e.g., that people can deal with the presented information due to the use of everyday language) and appraisal of novelty-complexity (e.g., due to the use of imagery). The previously discussed social presence elements are in line with Silvia's [29] suggestion to use imagery (e.g., human images) and everyday language (e.g., testimonials) to positively affect appraisal of novelty-complexity and coping potential respectively, resulting in increased interest. Moreover, increased interest results in spending more time reading information on a topic [31]. An initial hypothesis would therefore be that interest due to adding social presence elements increases website use (Hypothesis 3a). However, the increased complexity due to adding social presence elements can also have a negative impact. Previous studies demonstrated that complexity has a negative effect on attitude towards a website and therewith on intention to use [32], [33], pleading for simplicity of websites [34]. An alternative hypothesis would therefore be that adding social presence elements decreases website use because of increased complexity (Hypothesis 3b).

### Website

A website about Hepatitis A, B, and C virus (HAV, HBV, HCV) infections was used to test the effect of adding social presence elements on website use and the working mechanisms behind this possible effect. These infections all affect the liver, but there are differences in terms of mode of transmission, consequences, and prevention. Besides the home page, the website consisted of four pages per Hepatitis virus. The first page introduced the virus briefly and the other three pages outlined transmission, consequences, and prevention respectively. This resulted in a total of twelve pages of website content (for all virus types) besides the home page. The content for these pages was based on information from the Dutch National Hepatitis Centre. The content was text-based, purely informative, non-tailored, and very brief (i.e., 5–10 lines of text per page). Use of this website has been proven to result in increasing practical knowledge regarding Hepatitis [25], which is currently very low in the Netherlands [35]. The experimental website for this study was created by adding social presence elements to the website previously used, which served as the control website in this study.

### Social presence elements

Social presence elements were based on previous studies and consisted of adding human images (of people varying in age and gender) [12] and testimonials [13] in which people shared their experiences and ideas regarding the information presented on the website with the visitor. The above social presence elements were added to the home page and three pages per virus type that outlined transmission, consequences, and prevention respectively. Therefore, a total number of ten pages contained social presence elements. The number of pages of both website versions were identical. Previous research proved that JPEG file sizes are a good proxy for complexity of web pages [36]. File sizes are not only related to complexity, but also to page size (i.e., resolution). Therefore, screenshots of pages to which social presence elements were added in the experimental website, were captured for both websites at the same resolution (1024×768 pixels). The screenshots of the experimental website were – according to the JPEG file sizes – more complex in comparison with the control website (218 vs 137 kB, *t*(18) = 22.42, *p*<.001), which is a presumption in Hypothesis 3b (i.e., adding social presence elements increases complexity).

### Preparatory study

The aim of the preparatory study was to assess whether social presence elements attract participants' attention, because this is a prerequisite for them to affect user perceptions and (intention to) use. A previous study – based on self-reports – found a positive relationship between attention and user perceptions [37]. Eye-tracking, however, is a more suitable method to assess the course of attention over time [38].

This preparatory study built upon previous studies that successfully applied eye-tracking with regard to perceptions of health-related information [39] and websites [12]. There is general agreement on the strong association between eye movements and attention [40]. The idea behind eye-tracking is that saccadic eye movements are associated with shifts in attention. Fixations refer to the periods between saccadic eye movements in which gaze is held almost stationary.

## Methods

### Participants

Participants were a convenience sample of undergraduate students that received course credits for their participation in this preparatory study. Participants were screened to ensure normal or corrected vision, and those wearing hard contact lenses or eyeglasses were excluded from participation, because this can result in difficulties in capturing eye movements. A total of 24 participants were randomized (16 women, 8 men). Mean age of the sample was 20.1 years (*SD* = 1.8).

### Design and procedure

After explaining the study procedure and signing informed consent, participants were seated approximately 25 inches from a computer screen. The laboratory room was artificially lit and there was no influx of sunlight. First, the eye-tracker system (more detailed information in the Apparatus section) was calibrated. Subsequently, participants were told that we were interested in their opinion to improve a website and that they could freely explore this website. Participants were randomly assigned to two conditions. The control condition consisted of the existing website as used in a previous study [25]. The experimental condition added social presence elements to this website (as described above). A brief questionnaire had to be completed immediately following exposure to the website and consisted of measures regarding interest and perceived social presence as a manipulation check. It was stressed that there were no right or wrong answers.

### Ethics Statement

The Simon Fraser University Research Ethics Board granted ethical approval (file number: 2012s0168).

### Apparatus

The eye-tracker system used was Applied Science Laboratories Model 504 with head tracking integration. Eye movements were processed using an autofocus eye camera mounted on a optics mechanism (max. 100° pan, 25° tilt), which was positioned under the computer screen. The camera has eye illumination that consists of a ring of near-infrared light-emitting diodes. The sampling rate was 60 Hz and the measurement error was <1.0°. Participants wore a headband with a mounted sensor, which allowed tracking of head movements without loss of eye image. This permitted participants to move their heads in a relatively natural manner. Gaze Tracker software was used to capture and process eye-tracking data (measures are explained in the following section).

### Measurements

The following measurements were used within the preparatory study.

#### Attention

**Figure 1:**
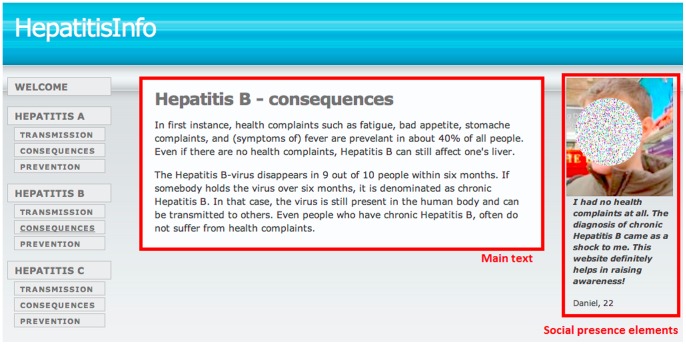
Example of lookzones on a page (image has been blurred to ensure anonymity).

Attention is assessed by the number of fixations and total fixation duration in milliseconds (ms) within each area of interest on the website as defined by rectangular regions called lookzones. There were two lookzones on each page; the main text and the social presence elements (Figure 1). The main text lookzone was similar for both conditions. The threshold for minimal duration for a fixation was set at 50 ms [40].

#### Interest

In line with Turner and Silvia [30], two items measured interest (uninteresting-interesting and boring-engaging; alpha = .72) on a 7-point Likert scale.

#### Perceived social presence

Five items (e.g., “There is a sense of human warmth in the website”; alpha = .87) were used to measure perceived social presence as a manipulation check [12], [41]. Items were answered on a 7-point Likert scale ranging from (1) ‘strongly disagree’ to (7) ‘strongly agree’.

### Analyses

Data from participants who encountered technical problems (e.g., difficulties in capturing eye movements) or artifacts (e.g., too many blinks) resulting in >25% missing eye-tracking data were excluded from the analyses regarding attention (*n* = 5). Mixed models were used to test for differences in the measures of attention, using page as a within-subject factor and condition as a between-subject factor. Two-tailed effect sizes (i.e., Cohen's d), given the means, standard deviations, and sample sizes per condition, were used to test differences between conditions regarding interest and perceived social presence. Effect sizes were divided into the following five levels: trivial (Cohen's d≤.2), small (>.2), moderate (>.5), large (>.8), and very large (>1.3) [42].

## Results

**Figure 2:**
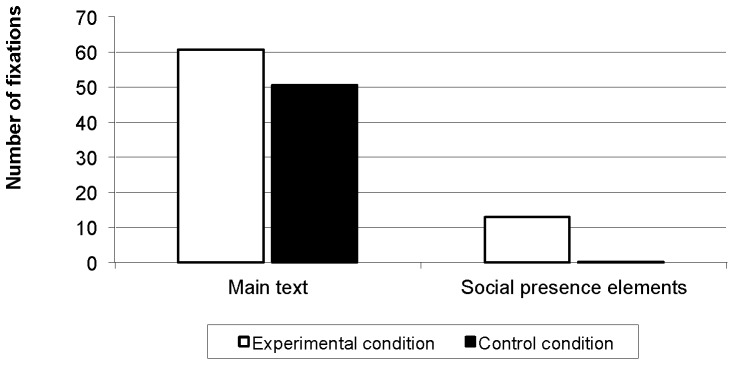
Number of fixations per page.

**Figure 3:**
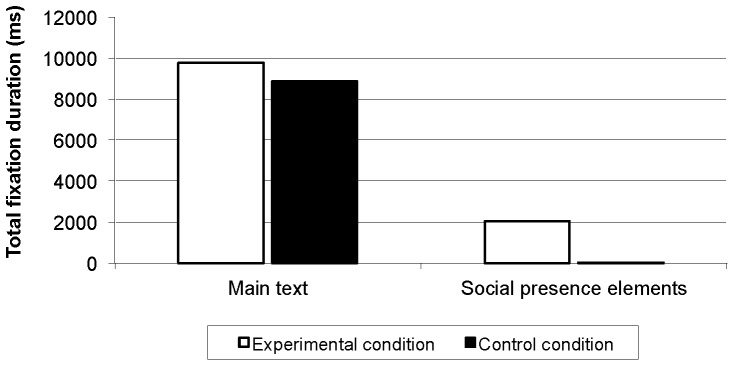
Total fixation duration (ms) per page.

Participants in the experimental condition focused on the social presence elements, both in terms of frequency (*F*(1, 98) = 40.34, *p*<.001) and duration (*F*(1, 88) = 39.99, *p*<.001), while participants in the control condition paid hardly any attention to these lookzones. There were, however, no differences between conditions in terms of frequency (*F*(1, 101) = 1.59, *p* = .21) and duration (*F*(1, 90) = 0.13, *p* = .72) of fixations on the main text. Figures 2 and 3 indicate that participants in the experimental condition were attracted by the social presence elements, but this did not affect their attention to the main text. Participants in the experimental condition scored higher on interest (*M* = 4.7 vs. 4.2, Cohen's d = 0.32) and perceived social presence (*M* = 4.6 vs. 3.1, Cohen's d = 1.22) in comparison with the control condition. These effects can be classified as small and very large respectively.

### Main study

The preparatory study demonstrated that the social presence elements attract participants' attention and increase perceived social presence, without decreasing attention for the main text. The aim of the main study was to test the proposed conceptual model and associated hypotheses.

## Methods

### Participants

Participants were recruited through a research panel of a Dutch Internet research agency; therefore, they could be considered computer literate. From this panel, a stratified sample of 982 potential participants was invited to participate in a study about Hepatitis through email. Informed consent was obtained online, which is the regular procedure for this research panel. This sample was representative of the Dutch Internet population above 18 years, taking into account gender, age, and level of education.

### Design and procedure

**Figure 4:**
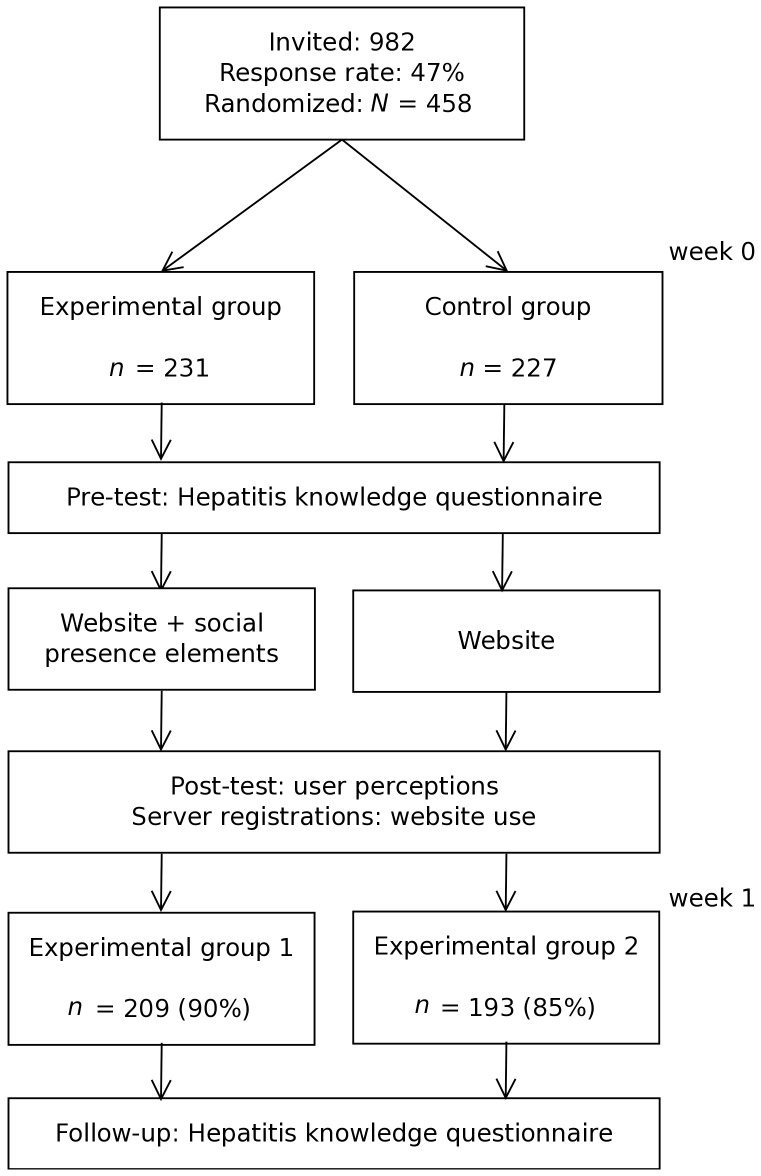
Flowchart of study design and attrition.

Participants were randomly assigned to either the control condition or the experimental condition (Figure 4). Both conditions were similar to those in the preparatory study. The pre-test (i.e., before being exposed to the website) consisted of a Hepatitis knowledge questionnaire. After the pre-test, participants were directed to their assigned version of the website. Participants were asked to base their opinion about the website on their first impression and were told they could freely explore the website until they started completing the post-test (immediately after visiting the website). The objective was not to force participants to thoroughly study the website, but to mimic a real-life situation in which time and opportunity to invest in the website is limited [4]. The post-test consisted of measures regarding user perceptions and perceived social presence as a manipulation check. For these measures, it was stressed that there are no right or wrong answers. One week later, participants were invited to complete the follow-up measure, which was the same as the pre-test (i.e., the Hepatitis knowledge questionnaire). The follow-up measure was added to test whether participants did not only click through the website but actually processed and memorized its content (i.e., by looking at both within- and between-group differences regarding Hepatitis knowledge over time).

Participants received an incentive (i.e., credit points for panel members) to participate in the study, which represented a value of € 2.50. Panel members can save credit points over time, which can be exchanged for online vouchers valid in several stores in the Netherlands. Relevant ethical safeguards were met with regard to the participant confidentiality and consent.

### Measurements

The following measurements were used within the main study.

#### Hepatitis knowledge questionnaire

Fifteen true/false items (including a ‘don't know’ option) about transmission, consequences, and prevention of HAV, HBV and HCV infections were used to assess Hepatitis knowledge. The sum score of all correctly answered items was used in the analyses. The correct answer to these items was available on the website. The items concerned practical knowledge and were used in a previous study [35].

#### Website use

Server registrations were used to assess website use [43], [44] which was operationalized by the number of pages visited (ranging from 0 to 12). Furthermore, time on the website was tracked to detect whether participants were simply clicking from one page to the other and therefore artificially boosting the number of pages visited.

#### User perceptions

Sets of three items each were used to measure efficiency (e.g., “I was able to access the information quickly on this website”; alpha = .97), effectiveness (e.g., “The website provided me with relevant information about …”; alpha = .92), enjoyment (e.g., “I found my visit to this website enjoyable”; alpha = .97), and active trust (e.g., “I would act upon the information presented on this website if needed”; alpha = .89). Items were answered on a 7-point Likert scale ranging from (1) ‘strongly disagree’ to (7) ‘strongly agree’. These measures were previously used and validated in the Dutch language [24]. Similar to the preparatory study, two items were used to measure interest (alpha = .82).

#### Perceived social presence

Similar to the preparatory study, five items (alpha = .95) were used to measure perceived social presence as a manipulation check.

## Analyses

First, independent samples t-tests and chi-square tests were conducted to test whether there was selective dropout or differences in dropout between conditions. Subsequently, independent-samples t-test were conducted to test for differences between conditions in terms of perceived social presence and both measures of website use. For this analysis, time on website was log-transformed to meet the assumption of normality. A repeated measures analysis of variance (ANOVA) was conducted to test for differences in Hepatitis knowledge between conditions over time. Multivariate ANOVAs were conducted to test for differences in user perceptions between conditions. All these analyses were conducted using Predictive Analytics SoftWare Statistics 18.0 (International Business Machines Corporation, Armonk, NY).

Second, using Mplus 5 (Muthén & Muthén, Los Angeles, CA), structural equation models (SEMs) using all available data were constructed to test the hypothesized conceptual model. Website use—a latent construct made up from number of pages visited and time on the website—was regressed on efficiency, effectiveness, enjoyment, interest, and active trust, which were all latent constructs using the items described above. Active trust was regressed on efficiency, effectiveness, and enjoyment. Effectiveness, enjoyment, and interest were regressed on social presence. Subsequently, (1) non-significant paths were left out of the conceptual model for the sake of parsimony, and (2) additional paths were added to the conceptual model based on significant modification indices. The latter was done to explore whether unanticipated relationships might explain variance in website use (which was not the case). A level of significance of *p*<.05 was used for the relationships within the model. Model fit indices used were the comparative fit index (CFI), the Tucker-Lewis index (TLI), the root mean square error of approximation (RMSEA), and the standardized root mean square residual (SRMR). Both CFI and TLI are goodness-of-fit indices where larger values signal better fit. Values over .95 indicate close fit. The RMSEA and SRMR are goodness-of-fit indices where larger values signal worse fit. Indicators of close fit are, respectively, RMSEA ≤.05 and SRMR ≤.09 [45], [46].

## Results

### Participants

Of those 982 potential participants that were invited, 458 participated in the study (47%). Half of the participants were female and the mean age of participants was 49 years (*SD* = 16). Of the participants, 30% had a low level of highest completed education (equivalent to primary school/junior high school), 38% an intermediate level (equivalent to senior high school/junior college), and 32% a high level (equivalent to college/university). Those 458 that participated were invited to complete the follow-up measure and 402 of them did so (88%). There was no selective dropout regarding gender (χ2 (1, N = 458) = 0.3, *p* = .57), level of education (χ2 = 5.0, *p* = .08), or Hepatitis knowledge at baseline (*t*(456) = 1.83, *p* = .07). Furthermore, dropout did not differ between the two conditions (χ2 (1, N = 458) = 3.2, *p* = .08). Those who dropped out, however, were younger (*M* = 43 vs 50 years, (*t*(456) = 3.19, *p* = .002).

### Manipulation check

Participants in the experimental condition scored higher on perceived social presence than the control condition (*M* = 4.9 vs. 4.4, *t*(456) = 4.11, *p*<.001, Cohen's d = 0.38), which indicates that the manipulation did succeed. This effect can be classified as small.

### Website use and Hepatitis knowledge

Participants in the experimental condition did not differ in website use in comparison with the control condition; neither in terms of the number of pages visited, nor in terms of time on the website (Table 1). Effect sizes were trivial. Thus, there is no support for Hypothesis 1. The increase in Hepatitis knowledge between pre-test and post-test did not differ between both conditions (*F*(1, 400) = 0.72, *p* = .40), but there was a significant increase regarding Hepatitis knowledge between pre-test and post-test across conditions (*M* = 6.4 vs. 8.3, *F*(1,400) = 172.98, *p*<.001, Cohen's d = 0.65), which can be defined as a moderate effect size.

**Table 1:** Differences in website use between conditions.

Variable	Experimental condition		Control condition		*t*	*p*	Cohen's d
	M	SD	M	SD			
Number of pages (0–12)	7.5	4.0	8.0	3.8	1.44	.15	0.14
Time on website (sec)	151.0	117.4	150.8	107.8	0.01	.99	0.00

### User perceptions

**Figure 5:**
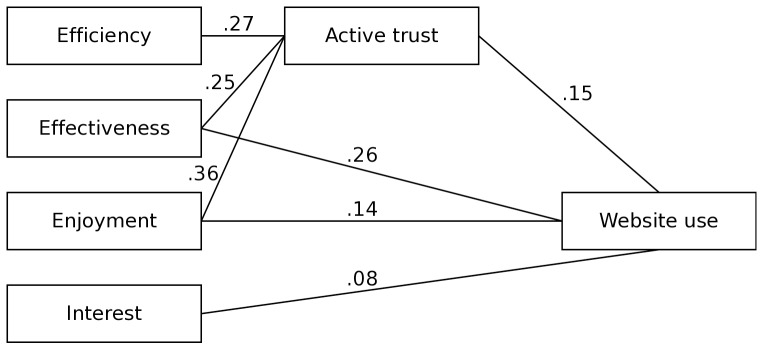
Final model including standardized betas of significant paths within the model.

There were no differences between conditions in terms of efficiency (*F*(1, 456) = 0.84, *p* = .36), effectiveness (*F*(1, 456) = 0.03, *p* = .87), enjoyment (*F*(1, 456) = 0.08, *p* = .78), active trust (*F*(1, 456) = 0.03, *p* = .87) and interest (*F*(1, 456) = 0.07, *p* = .80). Figure 5 illustrates the final structural equation model testing the associations between user perceptions and website use across conditions, because there were no differences on both measures of website use. The final model is mostly in line with Hypothesis 2: Efficiency (*M* = 6.5, *SD* = 0.84), effectiveness (*M* = 6.4, *SD* = 0.87), and enjoyment (*M* = 5.6, *SD* = 1.21) were positively associated with website use and these associations were partly mediated by active trust (*M* = 5.8, *SD* = 1.23). The association between efficiency and website use, however, was fully mediated by active trust. Since there were no differences between conditions in terms of website use, there is neither support for Hypothesis 3a nor for Hypothesis 3b. Interest (*M* = 5.9, *SD* = 1.06), however, was positively associated with and contributed to explaining variance in website use besides efficiency, effectiveness, enjoyment, and active trust. The explained variance (R^2^) of the number of pages visited and time on the website is .63 and .51 respectively. The CFI and TLI are .98 and .97, respectively; RMSEA and SRMR are .06 and .04, respectively. All of these fit indices indicate a close fit for the final model.

## Discussion

The key finding of the current study is that adding social presence elements did not affect actual use of an intervention website within a public health context. A possible explanation for this lack of effect can be derived from the results of the preparatory study: Even though the social presence elements attract attention, the frequency and duration of fixations on these elements was limited in comparison with the fixations on the main text. If the findings of the preparatory study are extrapolated to the main study, then this might explain the limited impact of social presence elements on actual website use.

It could be that people on a health-related website are mainly interested in the actual information it provides (i.e., the main text instead of social presence elements), which is mostly related to the utilitarian value of a website [47]. To date, however, the use of social presence elements has only been tested on more hedonically oriented websites (such as shopping for clothing) [14], [48]. It might be that social presence is more important on hedonically oriented websites in comparison with health-related websites, which are more utilitarian in nature. Even within the e-commerce context, there are differences between products: A previous study compared clothing (a more hedonic product for which consumers seek fun and entertaining shopping experiences) and headphones (a more utilitarian product for which consumers primarily seek detailed product information). Unlike clothing, higher levels of social presence on websites selling headphones did not have a positive effect on attitudinal antecedents [49]. In line with this, Nusair, Yoon, and Parsa [50] found that motivation variables, utilitarian and hedonic, moderate the effect of the web quality dimensions on satisfaction.

There are two other points that need to be raised while interpreting the findings of this study. First, there was a difference in effect size regarding perceived social presence, which was very large in the preparatory study, but only small in the main study. A speculative explanation could be that the sample of the preparatory study was much younger and might therefore be more open to social presence elements on websites. It is in our opinion more likely, however, that this is indicative of the difference between the lab setting in which participants had to look at the website in a more controlled environment versus the main study in which a real-life situation was mimicked. In the latter case, participants might pay only limited attention to a website [51], especially when added elements increase its complexity [33]. Second, this is the first study to investigate the effect of adding social presence elements on *actual* use instead of only *intention* to use or *attitude* towards a website. Although intention and attitude are important predictors of behavior [15], the intention-behavior gap is a well-known phenomenon in behavioral research: intentions account for 28% of the variance in behavior in prospective studies [52].

The explained variance of both behavioral measures in the current study (i.e., the number of pages visited and time on the website) was high and there was a close fit for the final model. In other words, user perceptions are appropriate predictors for explaining use of an intervention website in a public health context. Moreover, the relationships between these user perceptions and website use are comparable to the few other studies that are available within the field of attrition science regarding Internet-delivered interventions [24], [25]. The current study extends previous studies by including a measure of *interest* and demonstrates its added value besides the other perceptions. This is in line with the theoretical assumption of Silvia [29] that interest and enjoyment differ in critical ways and contributes to highly needed theory development within the field of attrition science [3].

A possible limitation of the study at hand is that participants in the main study were highly interested in the subject (i.e., Hepatitis), since they agree to participate in a study about this topic. Although participants perceived the website to be interesting (after being exposed to it), the low scores regarding Hepatitis knowledge at pre-test do not provide support for a possible selection bias regarding participants that were highly interested in the subject before the study. The increase regarding Hepatitis knowledge one week later is very positive, especially since participants were not necessarily looking for information on Hepatitis. The latter is essential in primary prevention websites aimed at the general public.

Future research needs to focus on characteristics of social presence elements that might affect their impact. The human images of people in the current study, for example, varied in age and gender. A previous study on Product Recommendation Agents (PRA; a decision support system that helps consumers to gather, screen, and evaluate available product information on the Internet), however, found that ethnicity-matched PRAs scored higher on perceived social presence, enjoyment, and effectiveness [53]. Another study revealed that PRAs with higher personalization also elicited more trust [54]. Personalization is one of the strategies, besides feedback and content matching, that is part of tailoring: creating communications individualized for their receivers, with the expectation that this individualization will lead to larger intended effects of these communications [55]. It might be that social presence elements on intervention websites in a public health context need to be tailored – a commonly used and effective method for the actual information on intervention websites [56]. Furthermore, the website used in this study was limited to providing knowledge about Hepatitis. It is worthwhile to investigate whether social presence elements may be more effective in increasing website use regarding intervention websites that are aimed at demonstrating and practicing skills (e.g., using role models) or reducing stigma that may be related to health behavior.

Another possible avenue for future research is whether or not cultural differences might be in play. Based on Hofstede's cultural value dimensions [57], a previous study revealed that the impact of pleasure on online behavior was higher for Canadian website visitors, while the impact of dominance was higher for Chinese website visitors [58]. To our knowledge, only one study has made a cross-cultural comparison of the impact of social presence elements, but this was limited to the impact user perceptions and actual use was not taken into account [59]. Hence, this is a nascent area worthy of additional investigation.
